# ESMO and WHO: 14 years of working in partnership on cancer control

**DOI:** 10.1136/esmoopen-2015-000012

**Published:** 2016-05-26

**Authors:** Andreas Ullrich, Fortunato Ciardiello, Gracemarie Bricalli, Nathan I Cherny, Alexandru Eniu

**Affiliations:** 1 World Health Organization, Geneva, Switzerland; 2 Second University of Naples, Naples, Italy; 3 European Society for Medical Oncology, Lugano, Switzerland; 4 Cancer Pain and Palliative Medicine Service, Department of Medical Oncology, Shaare Zedek Medical Center, Jerusalem, Israel; 5 Department of Breast Tumors, Cancer Institute Ion Chiricuta Cluj-Napoca, Romania

**Keywords:** ESMO

## Introduction

Since 2002, WHO and ESMO's proven track record of making progress to bridge the world of clinical oncology with the WHO's cancer control policies has kept cancer high on the world's political agenda. In 2013, after 10 years of working in partnership, ESMO was granted ‘official relations status’ with the WHO, which officially establishes a working relationship of an NGO with WHO. Such a relationship requires regular approval of 3-year work plans by the WHO Executive Board. The work plans harness the very distinct but complementary roles and responsibilities of both partners. Whereas ESMO is one of the leading international professional organisations for specialised physicians who are treating patients with cancer, WHO's cancer control policies are the reference for governments in their efforts to develop national cancer plans and a care delivery system for patients with cancer. By focusing on developing functional and comprehensive cancer plans, and a well-functioning healthcare system, WHO and ESMO seek to implement the WHO cancer management policies, facilitate the work of oncologists, and improve the outcomes of patients with cancer.

## Guiding principles for the partnership of WHO with NGOs (professional organisations)

It is stated in the WHO ‘Basic Document’^1^ that “the objectives of WHO's collaboration with NGOs are to promote the policies, strategies and programmes derived from the decisions of the WHO governing bodies,” which means, for example, decisions of the annual World Health Assembly. The WHO's cancer control policies and guidance are derived from a WHO Resolution on Cancer Control (The 58th World Health Assembly resolution on cancer prevention and control^2^) and are an integral part of the 2014 World Health Assembly Action Plan on Non-Communicable Disease (NCDs).^3^ It is important to note that WHO classifies cancer within the broader context of NCDs, that is, diseases which are generally considered non-infective and non-transmittable.

There are two strategies underpinning the collaboration between WHO and ESMO related to NCDs: focus on cancer prevention and control, and health system strengthening for cancer care.

## National cancer plans

A national cancer plan, according to the WHO standards, covers the three main policy areas of (1) cancer prevention to reduce the burden of cancer incidence; (2) national policies and strategies in early diagnosis and screening to reduce the proportion of cancers detected at advanced stages and (3) well-resourced healthcare delivery systems tailored to the needs of cancer management and care, including palliative care.

## Unmet needs in cancer control

Although many countries have begun to develop national cancer plans, as outlined in chapter 6.2 of the 2014 World Cancer Report,^4^ only a minority have implemented effective care delivery systems. In an ideal world, healthcare providers would be enabled by national healthcare systems to provide every patient with cancer—no matter where the patient lives—access to the best possible treatment, so that lives are saved and suffering reduced. However, there are tremendous inequalities in access to cancer treatment worldwide because only a minority of patients—mostly those living in high-income countries—are accessing treatment according to evidence-based clinical practice guidelines. The majority of patients with cancer do not have any, or only insufficient, access to treatment and care. More than 80% of the 12 million new patients with cancer per year worldwide (World Cancer Report 2014^4^) are living in low-income and middle-income countries where care delivery systems are ill-prepared to provide adequate cure and care. In addition, inequalities also exist in high-income countries. Indicators for these inequalities in access of care are differences in survival within and between countries.^5^ However, there is little and inadequate information about the quantitative aspects of unmet needs, such as shortages of medicines in daily practice, lack of adequate resources and medical devices, and the number of trained physicians available in each country, not to mention the underlying reasons for the existing gaps.

In 2011, to address unmet needs in cancer as well as other chronic diseases, the UN General Assembly held a UN High-Level Meeting on the Prevention and Control of NCDs in New York. At this meeting, Heads of State agreed on a UN Political Declaration^6^ which urged global prioritisation of cancer control and other NCDs, particularly in low-income and middle-income countries.

The European Union also issued a European Parliament Resolution^7^ calling for the EU and its Member States to actively implement the UN Political Declaration.

WHO was charged to develop a plan to implement the UN Political Declaration and to present it for adoption by the WHO Member States at the 2012 World Health Assembly.

During the 65th World Health Assembly in May 2012, the WHO Member States unanimously agreed to globally reduce preventable deaths from NCDs by 25% by 2025. For cancer, this would mean saving at least 1.5 million lives every year.

A year later, during the 66th World Health Assembly in May 2013, the WHO Member States unanimously adopted a three-part omnibus resolution outlining how they planned to achieve this 25% reduction in the global burden of NCDs by 2025. The three parts of the omnibus resolution that was approved are:

1. Adoption of a Global Monitoring Framework for NCDs^8^ establishing the goals and targets to be achieved and monitored;
2. Endorsement of the Global Action Plan for the Prevention and Control of NCDs 2013–2020^9^ outlining the action steps;
3. Agreement to establish a Global Coordination Mechanism^10^ to implement the plan at the national level in collaboration with other organisations.

ESMO delivered an official statement on the WHO Global Action Plan on NCDs at the 2013 World Health Assembly. It resulted in the creation of a ‘Global Cancer Task Force’ under ESMO's leadership. The members of the Task Force joined forces to collect data on the barriers to the availability of opioids for cancer pain management worldwide. The data collected was the first of its kind and served as background information for the 2014 World Health Assembly Palliative Care Resolution. The data helped Member States to see the urgency of the issue and to unanimously adopt the Resolution. The ESMO-led survey provides data on a country-by-country basis accompanied by 10 recommendations^11^ on how governments can reduce existing barriers to access to opioids.

## Strengthening the healthcare delivery system for cancer

In accordance with the WHO's conceptual framework for strengthening health care systems, an effective health care delivery system for cancer management and palliative care requires the establishment of a series of “building blocks” to enable access to care. This includes (1) that a sufficient number of well-trained oncologists and other specialists in cancer management such as surgeons and radiotherapists are available, (2) that national clinical practice guidelines are in place, (3) that medicines and technologies for cancer diagnosis and treatment are available for all patients in need (4) that there are monitoring systems in place including population-based cancer registries which provide the necessary data for planning and implementation of the cancer care system, (5) leadership and governance by Ministries of Health and (6) financing of the best possible public health service delivery.

Supporting these six building blocks are WHO programmes on essential medicines and technologies which include anti-neoplastic medicines and essential medication for cancer pain management and palliative care.

To assure the translation of WHO's cancer control policies into national action, WHO has country offices at the national level. Governments are encouraged to develop national cancer plans together with all stakeholders, including professional organisations, patient groups and academia. It is important that national stakeholders facilitate, catalyse and urge governments to incorporate the WHO recommendations into their national policies.

As previously mentioned, an important component of healthcare delivery is the proper training of clinicians. Professional organisations such as ESMO, and the knowledge networks that they create, allow clinicians worldwide to remain up to date on cutting-edge advances and research in order to treat patients according to evidence-based standards such as the ESMO Clinical Practice Guidelines.^12^

A healthcare system also requires an adequate workforce of physicians trained according to specific curricula. The ESMO-ASCO Recommendations for a Global Curriculum in Medical Oncology^13^ are an example of how ESMO is contributing on a global scale to prepare medical oncologists for the very challenging job of administering the broad spectrum of anticancer treatment regimens to patients safely and effectively.

## WHO–ESMO collaboration in the context of global and national cancer control policies

WHO has official relations with several NGOs working in the field of cancer control. WHO's collaboration with ESMO is based on ESMO's ‘official relations status’ and focuses on the implementation of WHO policies at the national level related to the clinical care of cancer patients. There are three key elements on which the work plan is based: (1) promotion of WHO's Model List of Essential Medicines, with its chapter on antineoplastic medicines and palliative care, (2) support for the WHO NCD and cancer control policies; and (3) dissemination of the WHO's cancer prevention and control strategies within ESMO's professional network.

## ESMO–WHO work plan objectives

1.  Support the WHO in its role to improve access to cancer treatment and essential medicines, by providing relevant worldwide data to support WHO's Access to Controlled Medications, the WHO Cancer Control Programs and the WHO document ‘Ensuring balance in national policies on controlled substances: Guidance for availability and accessibility of controlled medicines’**.**

2.  Support the WHO's implementation of the United Nations' Political Declaration on NCDs for capacity building, cancer prevention, treatment and palliative care through identification of ESMO members in key Member States to participate in meetings, advocate on a national level with Ministries of Health, and provide professional medical oncology expertise to the WHO consultations.

3. Raise awareness in developing countries about cancer prevention messages based on the ‘European Code Against Cancer’ produced by the WHO's International Agency for Research on Cancer (IARC) and in support of the WHO 2008–2013 Action Plan for the Global Strategy for the Prevention and Control of NCDs; WHO Global Strategy on Diet, Physical Activity and Health; WHO Global Strategy to reduce the harmful use of alcohol, and the WHO Framework Convention on Tobacco Control.

4. Provide WHO with an international platform to inform oncology professionals about the WHO activities and highlight the results of joint partnership projects with ESMO.

### Current status of implementation of the work plan

#### 1. Access to opioids

The WHO Model List of Essential Medicines^14^ includes a separate category for ‘Medicines for Pain and Palliative Care’, developed together with the International Association of Hospice and Palliative Care (IAHPC). In 2010 and 2013, ESMO published the European^15^ and Global Opioid Policy Initiatives.^16^ The studies were done in partnership with over 20 societies and surveyed the 8 major barriers to patient access to opioids for use in pain management. They also provided countries with 10 recommendations to reduce those barriers.

The data collected supported the 2014 World Health Palliative Care Resolution^17^ and was instrumental in the united lobby of the international palliative care and oncology community for an action plan on controlled medicines at the April 2016 UN General Assembly Special Session on Drugs (UNGASS). The Belgian Ministry of Health replied to a letter from ESMO that it would use recommendations in its planning for the 2016 UNGASS meeting in order to improve patient access to pain. In fact the UNGASS Outcome document contains a section entitled ‘Operational recommendations on ensuring the availability of and access to controlled substances exclusively for medical and scientific purposes, while preventing their diversion’.

#### 2. Access to cancer medicines

The WHO Model List of Essential Medicines^14^ also includes in its chapter 8.2 ‘Cytotoxic and adjuvant medicines’^18^ 46 cancer medicines, of which 16 new medicines were added in 2015 thanks to an international team led by the Union for International Cancer Control (UICC), of which ESMO is a member. The WHO list was published shortly after the release of the preliminary results of the ESMO-led European^19^ and International Consortium Studies on the Availability and Accessibility of Cancer Medicines^20^ in 2014 and 2015.

The medicines on the WHO Model List of Essential Medicines^14^ serve as a recommendation for national governments to provide these medicines to their citizens free of charge or at affordable costs so that they are widely accessible.

A new tool developed by ESMO, the ESMO Magnitude of Clinical Benefit Scale,^21^ has been designed by ESMO to provide insight into the degree of added value that new medicines approved by the European Medicines Agency (EMA) provide in the curative and non-curative settings. Scoring of medicines using this tool depends on which clinical trial data were input for the evaluation process, meaning that the score can change over time as new data become available.

The goal of ESMO Magnitude of Clinical Benefit Scale is to signal those newly-approved medicines with a large magnitude of clinical benefit, which should be endorsed and reimbursed across Europe for rapid patient access, especially when these medicines are recommended through evidence-based standards set forth in the internationally recognised ESMO Clinical Practice Guidelines.^12^

ESMO provided the WHO Regional Office for the Eastern Mediterranean with data regarding the availability of anti-cancer medicines in the region in order to help them plan their cancer management policies. By providing this first-of-its-kind data, ESMO supports the WHO Global Action Plan target to achieve by the year 2025 80% availability of essential medicines on the WHO Model List of Essential Medicines.^14^ The publication of the ESMO data are expected to facilitate a meaningful debate on critical issues related to the availability of inexpensive and expensive cancer medicines and to promote positive change.

#### 3. Cancer prevention

ESMO and WHO seek to reduce the burden of cancer at its earliest stages through effective cancer prevention awareness and advocacy campaigns. The ESMO position paper on ‘The perspective and role of the medical oncologist in cancer prevention’^22^ supports WHO efforts on this topic and urges EU Member States to make cancer prevention a priority in order to reduce the burden of cancer among their citizens.

Designing cancer prevention projects based on WHO and IARC's ‘European Code Against Cancer’, ESMO and several partner societies produced a video, posters and a Cancer Prevention Advocacy Toolkit for Africa.^23^ Similar projects in Latin America and Asia are planned. In Europe, ESMO is a founding member of the European Chronic Disease Alliance^24^ which focuses on minimising common lifestyle risk factors across chronic diseases, including cancer. ESMO's cancer prevention initiatives support the WHO Global Action Plan target to reduce premature deaths from non-communicable diseases by 25% by 2025.

#### 4. ESMO's European and Global Public Policy Committees

ESMO works at the political level on issues related to the practice and profession of medical oncology. Its EU Policy and Global Policy Committees focus on a wide range of topics dealing with issues like cancer prevention, data protection, clinical trials, access to medicines, reference networks for rare cancers, and palliative care, to name a few.

By participating in key committees such as the European Medicines Agency's Human Scientific Committees’ Working Party with Healthcare Professionals’ Organisations and the European Network of Health Technology Assessment (EUnetHTA), ESMO is well positioned to provide input to discussions regarding cancer medicine evaluation and reimbursement.

As a member of the WHO Europe Expert Group on NCDs and the European Commission Expert Group on Cancer Control, ESMO is pleased to provide technical expertise and strategic input to WHO projects and planning - as well as to disseminate WHO documents to ESMO members worldwide.

ESMO's European and global networks engage ESMO National Representatives and partner societies to join in helping WHO implement its Global Action Plan for NCDs at the national level. Country workshops are planned to discuss with key stakeholders current needs and future actions.

## Conclusion

The complementary roles and functions of WHO and ESMO allow us to work side-by-side to improve the outcomes of cancer patients worldwide.

The data collected by ESMO highlights where the barriers are and where WHO efforts should be targeted to adjust health care delivery systems for better care. The Anti-neoplastic medicines survey gathered data to improve knowledge on cancer medicine shortages, because there is currently no comparable international data available. Once the manuscript is published, ESMO will work with national medical societies and WHO Regional and Country Offices to understand how best to tackle this issue.

In addition to contributing to the update of the 2015 WHO Model List of Essential Medicines,^14^ ESMO is also providing advice to the WHO Priority Technologies List. Building on these projects a next step will be an oncology workforce survey with the WHO Global Health Workforce Alliance. The survey will match the list of necessary cancer interventions to professional competencies required to deliver them. The information will support the WHO Global Strategy on Human Resources for Health aimed to inform governments about future workforce shortages, including those in oncology. It will encourage governments to align their national cancer plans with the WHO Global Cancer Plan, and assure priorities and actions are coherent across other national agencies and programs.

Joint promotion to governments worldwide of WHO policies and ESMO recommendations will save lives, increase quality of life, and reduce unnecessary suffering. For all of our joint projects ESMO and WHO are sincerely grateful to the partner societies, and to all ESMO members, whose expertise and commitment continue to allow us to raise awareness of crucial public policy issues across the globe.

We look forward to many more years of fruitful collaboration!
